# Children with disabilities lack access to nutrition, health and WASH services: A secondary data analysis

**DOI:** 10.1111/mcn.13642

**Published:** 2024-04-02

**Authors:** Isabel Rice, Charles Opondo, Lydia Nyesigomwe, Daniel Ekude, Julius Magezi, Andrew Kalanzi, Marko Kerac, Julia Hayes, Malia Robello, Sarah Halfman, Emily DeLacey

**Affiliations:** ^1^ Department of Population Health, London School of Hygiene & Tropical Medicine, Faculty of Epidemiology and Population Health University of London London UK; ^2^ Department of Medical Statistics, London School of Hygiene & Tropical Medicine, Faculty of Epidemiology and Population Health University of London London UK; ^3^ Holt International‐Uganda Office Kampala Uganda; ^4^ London School of Hygiene & Tropical Medicine, Centre for Maternal, Adolescent, Reproductive, & Child Health (MARCH) University of London London UK; ^5^ Holt International Eugene Oregon USA

**Keywords:** child nutrition, dietary adequacy, disability, health access, nutrition and health services, WASH

## Abstract

Malnutrition and disability are major global public health problems. Poor diets, inadequate access to nutrition/health services (NaHS), and poor water, sanitation and hygiene (WASH) all increase the risk of malnutrition and infection. This leads to poor health outcomes, including disability. To better understand the relationship between these factors, we explored access to NaHS and household WASH and dietary adequacy among households with and without children with disabilities in Uganda. We used cross‐sectional secondary data from 2021. Adjusted logistic regression was used to explore associations between disabilities, access to NaHS, WASH and dietary adequacy. Of the 6924 households, 4019 (57.9%) reported having access to necessary NaHS, with deworming and vaccination reported as both the most important and most difficult to access services. Access to services was lower for households with children with disabilities compared to those without, after adjusting for likely confounding factors (Odds ratio = 0.70; 95% CI 0.55–0.89, *p* = 0.003). There is evidence of an interaction between disability and WASH adequacy, with improved WASH adequacy associated with improved access to services, including for children with disabilities (interaction odds ratio = 1.12, 95% CI: 1.02–1.22, *p* = 0.012). The proportion of malnourished children was higher among households with children with disabilities than households without it (6.3% vs. 2.4% *p* < 0.001). There are concerning gaps in access to NaHS services in Uganda, with households with children with disabilities reporting worse access, particularly for those with low WASH adequacy. Improved and inclusive access to NaHS and WASH needs to be urgently prioritized, especially for children with disabilities.

## Key messages

- There is low access to essential nutrition and health services for households in Uganda: access barriers need to be examined and minimized.
- Children with disabilities have particularly poor access to key nutrition and health services. Clean water, sanitation and good hygiene for children with disabilities are associated with improved access to nutrition and health services.

## Graphical abstract

Children with disabilities lack access to essential nutrition, health and WASH services.

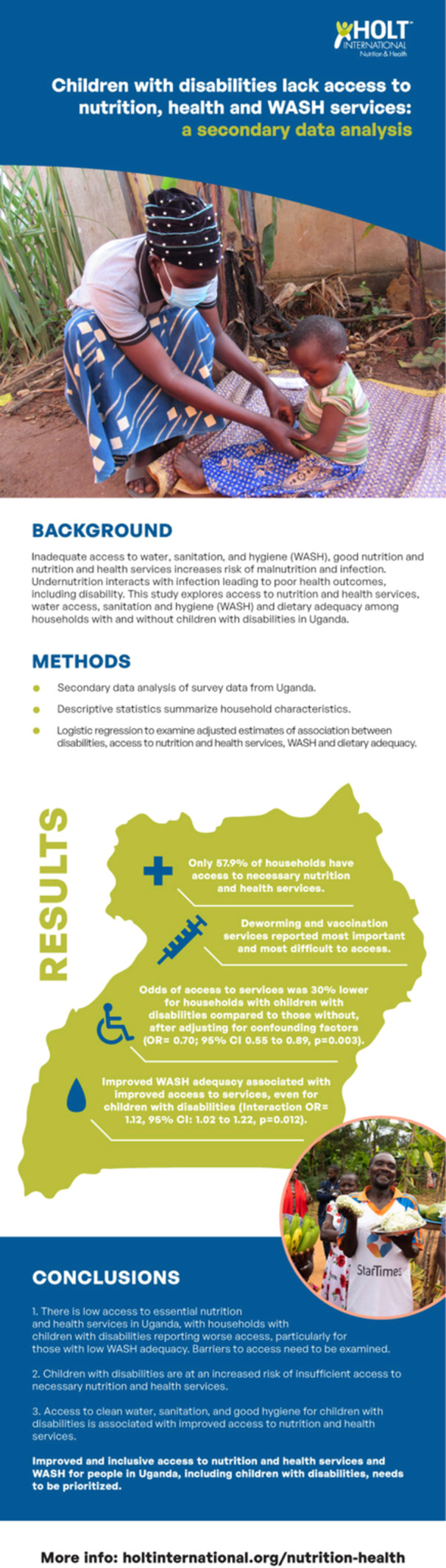

## 1 INTRODUCTION

An estimated 45% of deaths in children younger than 5 years old globally have undernutrition as an underlying or contributory cause. Millions more are affected by infections and other morbidity, which can result in long‐term impacts to children's health and development (Black et al., 2013; Grey et al., 2021; Lelijveld et al., 2016). Despite progress towards the 2030 Sustainable Development Goals (SDG), some 22% (149.2 million) of children globally are stunted, 6.7% (45.4 million) are wasted and 5.7% (38.9 million) are overweight (United Nations, 2022; Victora et al., 2021). While malnutrition and micronutrient deficiencies have decreased globally, a high burden of these conditions continues in low‐ and middle‐income countries (LMICs), including many in Sub‐Saharan Africa (SSA) (Onyango et al., 2019; Quamme & Iversen, 2022). Malnutrition, poor dietary diversity and limited healthcare access are all factors leading to increased incidence of infections, preventable deaths, chronic disease, impaired immune function, disability and impacted economic and educational outcomes (Black et al., 2013; Han et al., 2022; Tam et al., 2020). Infections can cause or worsen malnutrition by factors such as increased nutritional needs, gastrointestinal damage, malabsorption and reduced appetite—perpetuating a vicious infection/malnutrition cycle (Katona & Katona‐Apte, 2008). However, despite a high need for nutrition and health services (NaHS) in SSA, access is low (42.56%), particularly for women, children and people with low socioeconomic status, low levels of education, rural residence and those with disabilities (Tessema et al., 2022; World Health Organization, 2011). Specifically, access to Vitamin A supplementation, treatment for malnutrition, vaccination, HIV care and deworming remains low (Black et al., 2013). For example, only 59.4% of children under the age of 2 are reached by vitamin A supplementation coverage in SSA (Berde et al., 2019). Insufficient NaHS access often occurs in tandem with other inaccessible household needs such as nutritious food and clean drinking water (Black et al., 2013; Drammeh et al., 2019). Some 17 million individuals in SSA are food‐insecure, have poor water access, sanitation and hygiene (WASH) adding additional challenges to already stressed households and contributing to the high prevalence of malnutrition and micronutrient deficiencies (Drammeh et al., 2019; Masangcay et al., 2021). Globally, mortality and morbidity related to poor WASH is declining, with the exception of SSA where there is particularly low coverage of access to safe drinking water and sanitation (Fuente et al., 2020; World Health Organization [WHO], & United Nations Children's Fund [UNICEF], 2021). Widespread public health issues, climate change and other factors have further exacerbated existing gaps and barriers in access to health care in SSA, with many services closed or overwhelmed and households facing added financial barriers to access (Tessema et al., 2021).

Some people, such as those with disabilities, are at especially high risk of malnutrition and infections, which can exacerbate existing disabilities or cause disabilities (Groce et al., 2014; Rotenberg et al., 2024). Globally, over one billion people are disabled, of which 150 million are children—with 80% of these individuals living within LMICs (Hume‐Nixon & Kuper, 2018; United Nations Children's Fund, 2021; World Health Organization, 2011). Children with disabilities are nearly three times more likely to be underweight and twice as likely to be stunted and wasted than children without disabilities (Hume‐Nixon & Kuper, 2018; Rotenberg et al., 2024). Despite this, children with disabilities are often missing from malnutrition guidelines, protocols and government policies or initiatives (Engl et al., 2022; United Nations Children's Fund, 2021). The World Health Organization defines disability as, ‘The interaction between individuals with a health condition … and personal and environmental factors’, which indicates a person's experience of their disability is greatly impacted by the accessibility of their environment. Despite often having higher needs for services, those with disabilities frequently experience barriers, discrimination and stigma when accessing NaHS (Adugna et al., 2020; Groce et al., 2013; Hume‐Nixon & Kuper, 2018; World Health Organization, 2011). Some examples of barriers can include inaccessible health facilities or transportation, medical professionals or healthcare programmes not structured or trained to provide services for children with disabilities or stigma against disabilities resulting in children being unwelcome or deemed a lower priority for NaHS (Adugna et al., 2020; Groce et al., 2014). Difficulties accessing essential NaHS can further stress already insecure households with children with disabilities. Children with disabilities are at high risk of abandonment and are disproportionately present in institution‐based care (DeLacey et al., 2020, 2021).

Uganda is a key example of a country in SSA where malnutrition, infections, disability and inadequate access to NaHS remain influential factors in morbidity and mortality among children (Global Nutrition Report, 2021; Mawa, 2018). Approximately 7.5% of 5‐ to 17‐year‐olds and 3.5% of 2‐to 4‐year‐olds in Uganda have a disability, although this figure may be underestimated (United Nations Children's Fund [UNICEF], 2019). Uganda has the highest mortality rate from diarrhoeal infections in children younger than 5 years old in East Africa (22%) (Omona et al., 2020). The existing small body of evidence suggests people with disabilities in Uganda face multiple barriers to accessing NaHS and WASH services, however, there is limited information on access to NaHS, household WASH and dietary adequacy for children with disabilities (Adugna et al., 2020; Tessema et al., 2021, 2022).

### 1.1 Aim and objectives

The aim of this study is to inform improvements in future nutrition, health and WASH delivery for households in Uganda, with a particular focus on children with disabilities.

We will achieve this through three related objectives:

1. Describe demographics and general access to NaHS, household WASH status and dietary adequacy among households in the study population.
2. Examine access to NaHS for households with children with disabilities and without disabilities.
3. Explore WASH and dietary adequacy for households with children with disabilities and without disabilities.

## 2 METHODS

### 2.1 Study design

This is a secondary analysis of cross‐sectional survey data originally collected in 2021 by Holt International in Uganda. Holt International is an international child welfare nonprofit which provides support and services to orphaned and vulnerable children and their families in 15 countries (Holt International, 2022). Holt International has worked in Uganda since 2006 and provides NaHS services to children and families in three districts. Ethical approval was received from the London School of Hygiene and Tropical Medicine—(Reference: 27255) for this study.

We followed the STROBE reporting guidelines and the PECO framework, which outlines the population, exposure, comparator group and outcomes of interest (Table A1). (Mintzker et al., 2022; von Elm et al., 2008).

### 2.2 Setting

The survey took place in three districts in central Uganda; Mukono, Luwero and Wakiso, where Holt International operates NaHS (Supporting Information: S2). (Uganda Bureau of Statistics, 2020).

1. Wakiso District has a total population of 2,915,200—60% of these are younger than 20 years old and 1,534,200 (52.6%) are females.
2. Mukono District has a total population of 701,400 people and 60% of these are younger than 20 years old and 361,809 (51.6%) are females.
3. Luwero District has a total population of 523,600 people and 60% of these are younger than 20 years old and 262,700 (50.2%) are females.

Holt provides NaHS to rural communities who the Ugandan government previously identified as lacking access to health services. NaHS include medical checkups and treatment of illnesses for adults and children, immunization, deworming, supplementation, perinatal nutrition and breastfeeding support.

### 2.3 Participants

Eligibility criteria for participation in the household survey included all households within the sample districts with one or more children younger than 18 years old. An adult from each of the participating households responded to the survey on behalf of the household. The survey was conducted by 15 professional surveyors in Luwero, five in Mukono and five in Wakiso districts, who were supervised by five supervisors, and three team leaders.

### 2.4 Study size and sampling strategy

The household survey collected data from households participating in Holt's programmes (i.e., family strengthening programmes or sponsorship), as well as households in the districts not participating in Holt's programmes. The survey team contacted local community leaders for village registers of residents. These registers were used to determine numbers of residents for sampling. The sampling strategy utilised for recruiting non‐Holt supported households from across the three districts was systematic and unweighted by sampling every second household. Households participating in Holt programmes were purposely sampled from the same communities, with all households eligible for participation. Surveyors were trained in minimizing responder bias, measurement error, ethical practices in surveying, voluntary and informed participant consent, data protection, survey practices to respect cultural norms, practices and participant comfort. At the survey level, systematic sampling was used and community leaders were engaged to encourage all to take part, thereby minimizing response bias. If a family was absent during the survey, the surveyor would return the next day. Respondents self‐reported on behalf of their households. Mid‐upper arm circumference (MUAC) measurements of children were taken by a trained surveyor using coloured MUAC tapes for all children ages 6 months to 5 years old who were present at the time of the survey.

### 2.5 Survey questionnaire

The survey questionnaire was designed by Holt International as part of routine programming and data collection in Uganda (Supporting Information: S2). The survey focused on household access to NaHS and included questions regarding health access and needs, WASH, nutrition and dietary adequacy, as well as demographic information. This survey was conducted to inform and improve Holt's programming and services and provide information for scale‐up of NaHS in the area. Survey questions' responses included binary, categorical, free text or numerical variables. Disability status was self‐reported by respondents on behalf of their households. Households were asked if they had a child with a disability with a response of ‘yes’ or ‘no’. If yes, then additional questions on self‐reported disability type, impact to daily functioning, difficulties eating and others were asked of the respondent (Table A4). Other questions in the survey included how many meals a day a child received and their consumption of food from five food groups as reported by the respondent (Table A4).

### 2.6 Data analysis

**Figure A1:**
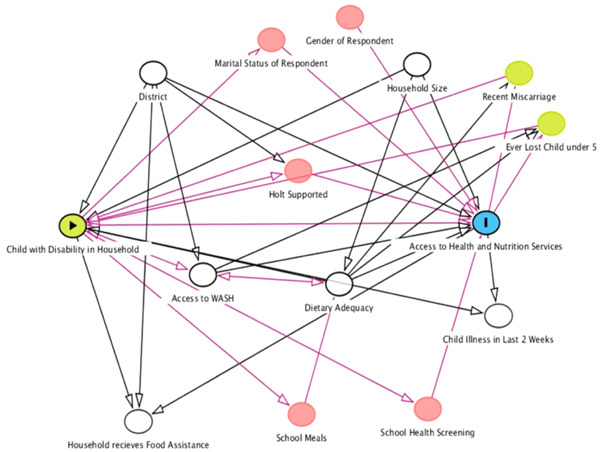
Directed acyclic graph exploring the relationship between child disability and access to nutrition and health services with other factors green circles indicate an ancestor of the exposure on the causal pathway. White circles indicate adjusted variables. Purple arrows indicate a causal pathway. Pink circles indicate an ancestor of both the exposure and outcome, thus on the causal pathway.

A deidentified, fully anonymized data set was used for analysis. A conceptual framework to understand potential confounders was created before analysis, with WASH and dietary adequacy selected as potential effect modifiers to be explored as proxy markers of poverty. A statistical analysis plan was created before analysis and guided the analysis process to reduce potential bias. STATA software (version SE 17) was used for all statistical analysis (StataCorp, 2017). Duplicates and outliers were explored and removed. Missingness of data was explored with a view to conducting a complete case analysis if missingness was low (<5%). The main exposure variable was households with children with disabilities, and the main outcome variable was self‐reported household access to NaHS. For the outcome, a binary variable was used based on the question, ‘Do you currently have access to all health services that you need?’, to which participants answered either ‘yes’ or ‘no’. A directed acyclic graph (DAG) was created to explore the association between the exposure (children with disabilities in the household) and outcome (access to NaHS) alongside potentially confounding factors to inform regression models (Figure A1).

A WASH adequacy score was generated based on the questionnaire to synthesize household WASH adequacy. The initial questionnaire asked nine questions relating to WASH adequacy (Supporting Information: S2). The survey question, ‘In the last one month, has there been a time when you did not have sufficient water for drinking?’, with a response of ‘yes’ or ‘no’, was taken as an overall indicator of WASH adequacy for use as a binary variable in analytical statistics. Additionally, a WASH adequacy score was created based on summing responses from some of the WASH‐related questions, with a higher score indicating greater adequacy of WASH services. The WASH adequacy score also took into account the safety of the water source, with bottled water and piped water receiving higher scores and open wells receiving the lowest score. To analyse dietary intake, binary ‘yes’ or ‘no’ variables were created if a child consumed three meals a day or if they consumed food from the five food groups measured over the previous 7 days. From these two variables, the dietary adequacy variable was generated based on if a ‘yes’ was reported for both of those variables (Table A5).

The survey included data on individual child MUAC measurements from participating households (Supporting Information: S2). These anthropometric measurements were analysed for children 6 months to 5 years old based on WHO guidance, with severe malnutrition classified as less than 11.5 cm, moderate malnutrition less than 12.5 cm, and within normal range being greater than or equal to 12.5 cm (World Health Organization, 2019). Data outliers were removed to correct for measurement error, based on a plausible range of 8.5–20.0 cm for children ages 6 months to 5 years. Children with disabilities were included in this measurement.

Descriptive statistics were used to summarise demographic characteristics of included children and households, with counts and proportions reported for each categorical variable. Count variables for household size and number of children per household were summarised as median, interquartile range and total range. Continuous variables were described using mean and standard deviation. Descriptive statistics were reported by household status; all households, those with children with disabilities and those without children with disabilities. *χ* ^2^ test of association (or Fisher's exact test where appropriate) and *t* tests were used to compare characteristics of households with and without children with disabilities.

Logistic regression was used to explore the association between a binary explanatory indicator of child disability within the household and a binary outcome indicator of household access to NaHS. Adjusted analysis included all potentially confounding factors identified from the DAG (Figure A1). Interactions were fitted between the main exposure and both WASH and dietary adequacy which were used as proxy indicators of poverty, to establish if the association between exposure and outcome differed by access to WASH services and nutrition. Interaction terms were tested using a likelihood ratio test.

### 2.7 Ethical statement

Ethical review and approval for this study was received from the London School of Hygiene and Tropical Medicine (Reference: 27255). Research was conducted in accordance with the ethical standards of the 1964 Declaration of Helsinki and its later amendments or comparable standards.

## 3 RESULTS

**Figure 1:**
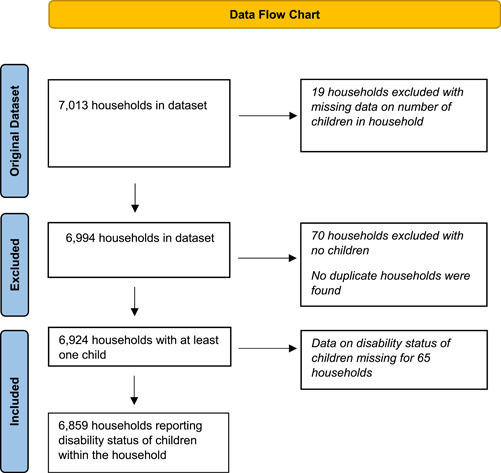
Data flow chart indicating the number of households included in the analysis.

A total of 12,260 non‐Holt‐supported households were indicated from the village registers, with a sample of 6130 participants for the survey. A total of 853 Holt‐supported households were recruited from purposive sampling and data was collected from 7013 households in total. The 70 households in the sample with no children were excluded, with a further 19 households removed due to missing data on the number of children in the household (Figure 1). Household identification numbers were explored for duplicates and none were found. This resulted in a sample of 6924 households reporting at least one child in the household included in the analysis (Figure 1).

Of the 6942 households, 1176 (16.9%) were from Mukono district, 4082 (58.8%) from Luwero and 6942 (24.3%) from Wakiso (Tables 1 and A4). Of the households surveyed, 567 (8.2%) were already participating in Holt programmes. Females made up 85.4% of respondents. Most respondents were married (5417 [78%]). Widows accounted for 386 (5.6%) of respondents with the highest proportion among households of children with disabilities (7.9%). Husband and wife‐headed households were most common (5292 [76.2%]).

**Table 1:** Demographic characteristics of households by households with and withoutded households were most commoded households were most commo children with disabilities.

Characteristic^a^	All households	Households with children with disabilities	Households without children with disabilities	*p* Value
*N* (%)	6942	305	6554	
Holt‐supported household				*p* = 0.202
Yes	567 (8.2)	31 (10.2)	533 (8.1)	
Missing	19 (0.3)			
Respondent gender				*p* = 0.456
Female	5929 (85.4)	256 (83.9)	5600 (85.4)	
Marital status of respondent				*p* = 0.032
Married	5417 (78)	220 (72.1)	5128 (78.2)	
Single	1139 (16.4)	61 (20)	1068 (16.3)	
Widow/widower	386 (5.6)	24 (7.9)	358 (5.5)	
Head of household				*p* = 0.154
Husband and wife	5292 (76.2)	220 (72.1)	5005 (76.4)	
Grandmother	669 (9.6)	38 (12.5)	624 (9.5)	
Female	981 (14.1)	47 (15.4)	925 (14.1)	
Number of people in household				
Median (IQR)	5 (4–6)	6 (4–8)	5 (4–6)	
Number of children in household				
Median (IQR)	3 (2–4)	4 (2–5)	3 (2–4)	
Household has attended Holt NaHS	*p* = 0.841			
Yes	644 (9.3)	27 (8.9)	614 (9.4)	
Missing	77 (1.1)			
Household visits health centre when sick				*p* = 0.100
Yes	6065 (87.4)	261 (85.6)	5797 (88.5)	
No	564 (8.1)	27 (8.9)	536 (8.18)	
Self‐medicate (uses traditional medicine or over the counter medicine)	236 (3.4)	17 (5.6)	218 (3.3)	
Missing	77 (1.1)			
Child had medical check last in year				
Yes	1710 (24.6)	75 (24.6)	1632 (24.9)	*p* = 0.946
Missing	77 (1.1)			
Access to all nutrition and health services needed				
Yes	4019 (57.9)	151 (49.5)	3863 (58.9)	*p* = 0.001
Missing	77 (1.1)			
Most important health services to household^b^				
Vitamin supplements	1650 (23.8)	64 (21)	1584 (24.2)	
Deworming	2409 (34.7)	100 (32.8)	2305 (35.2)	
Child growth screening	1523 (21.9)	75 (24.6)	1447 (22.1)	
Wellness check	1831 (26.4)	83 (27.2)	1747 (26.7)	
Vaccinations	2079 (30)	94 (30.8)	1982 (30.2)	
Prenatal care	1004 (14.5)	42 (13.8)	962 (14.7)	
Nutrition services	1567 (22.6)	72 (23.6)	1493 (22.8)	
Missing	1437 (20.7)			
Health services unable to access^b^				
Vitamin supplements	483 (7)	23 (7.5)	457 (7)	
Deworming	723 (10.4)	44 (14.4)	676 (10.3)	
Child growth screening	305 (4.4)	7 (2.3)	298 (4.5)	
Wellness check	561 (8.1)	28 (9.2)	533 (8.1)	
Vaccinations	827 (11.9)	57 (18.7)	769 (11.7)	
Prenatal care	197 (2.8)	8 (2.6)	189 (2.9)	
Nutrition services	354 (5.1)	11 (3.6)	342 (5.2)	

a All characteristics are self‐reported by the respondent on behalf of their household.

b Variables not mutually exclusive.

In total, 305 (4.4%) of households reported at least one child with a disability. Loss of a child younger than 5 years old was reported by 441 (6.4%) of households and 617 (8.9%) reported a miscarriage in the last 5 years, with households with children with disabilities reporting a higher proportion of miscarriage (15.4% vs. 8.6% *p* < 0.001) (Table A4).

The distribution of number of people and number of children in the household was similar across districts. The median number of people in each household was five (IQR 4–6), with a median of three children in each household (IQR 2–4) (Table 1). Missingness was negligible among demographic variables (<1.5%), most having no missing data.

Information on access to NaHS indicated insufficient access for households with and without children with disabilities (Table 1). Households with children with disabilities reported lower access to necessary health services than households without children with disabilities (49.5% vs. 58.9% *p* = 0.001). Only 644 (9.3%) of households had ever attended a Holt NaHS. Most households (6065 [87.4%]) reported visiting a health centre when they are sick. Households with children with disabilities had a higher prevalence of illness in the last two weeks, including diarrhea, skin infections, cough or fever, than those without children with disabilities (76.7% vs. 67.3%, *p* = 0.001) (Table A2).

The health service reported as most important was deworming (2409 [34.7%]), followed by vaccinations (2079 [30%]) and nutrition services (1567 [22.6%]). Prenatal care was deemed least important (1004 [14.5%]). Vaccinations (827 [11.9%]) followed by deworming (723 [10.4%]) were the most common services households reported as inaccessible, with a higher proportion of insufficient access among households with children with disabilities (57 [18.7%] and 44 [14.4%]). It was reported by 1710 (24.6%) that their children had a medical checkup in the last year and 1740 (25.2%) of households reported their children received health screenings at school.

Of the 6862 households that provided responses on child dietary adequacy for children older than 2 years old, 4488 (64%) of households reported children having three meals a day, with a slightly higher proportion among households without children with disabilities (65.7% vs. 60.6% *p* = 0.073) (Table 2, Table A2 and Table A3). Overall, 1196 households (17.4%) reported children meeting the criteria for dietary adequacy. Of the households with children with disabilities, only 44 (14.4%) reported dietary adequacy. MUAC was measured for 8357 children, of which 221 (2.6%) were malnourished (MUAC less than 12.5 cm). Households with children with disabilities were more malnourished than those without (28/442 [6.3%] vs. 190/7785 [2.4%] *p* < 0.001) (Table 2 and Table A2).

**Table 2:** Drinking water sufficiency, dietary adequacy, WASH adequacy and child nutrition status by household status.

Characteristic^a^	All households	Households with children with disabilities	Households without children with disabilities	*p* Value
*N* (%)	6942	305	6554	
Lacked sufficient drinking water last month				*p* = 0.888
Yes	1531 (22.1)	66 (21.6)	1465 (22.4)	
Missing	112 (1.6)			
WASH adequacy score^c^				*p* = 0.497
Mean (standard deviation)	11.8 (2.9)	11.6 (3.1)	11.8 (2.9)	
Median (IQR)	12 (10–14)	12 (10–14)	12 (10–14)	
Missing	83 (1.2)			
Children have three meals a day^a^				*p* = 0.073
Yes	4488 (64)	183 (60.6)	4264 (65.7)	
Missing	151 (2.2)			
Children achieve dietary adequacy^a^				*p* = 0.188
Yes	1196 (17.4)	44 (14.4)	1142 (17.4)	
Missing	151 (2.2)			
Child under 5 years of age is malnourished				*p* < 0.001
Yes	221/8357 (2.6)	28/442 (6.3)	190/7785 (2.4)	

^a^ At individual child level not household level.

^b^ Defined by a mid‐upper arm circumference (MUAC) less than 12.5 cm. (World Health Organization, 2019).

^c^ WASH adequacy score out of 20, compiled from responses to seven questions about WASH.

A total of 1531 (22.1%) households reported lacking sufficient drinking water in the last month. WASH adequacy score was approximately normally distributed (mean:11.8, SD:2.9), and the median score in all districts was 12 out of a possible total of 20 (IQR 10–14). Households with and without children with disabilities had similar WASH adequacy scores (Table 2, Table 4 and Table A5).

The unadjusted odds of reporting access to all NaHS needed was 32% lower (OR = 0.68; 95% CIs 0.54–0.86; *p* = 0.001) for households with children with disabilities compared to households without children with disabilities, indicating very strong evidence of an association (Table 3). The fully adjusted odds ratio (OR = 0.70; 95% CIs 0.55–0.89; *p* = 0.003) indicates households including children with disabilities had 30% lower odds of having access to NaHS than households with no children with disabilities, with very strong evidence of an association persisting after adjusting for sociodemographic characteristics, WASH and dietary adequacy.

**Table 3:** Crude and adjusted analysis of characteristics associated with access to nutrition and health services.

		Unadjusted odds ratio for access to nutrition and health services	Odds ratio adjusted for sociodemographic factors^a^	Odds ratio adjusted for sociodemographic factors, WASH and dietary adequacy^b^
Characteristics	Households with access to nutrition and health services total (%)	(95% CI) *p* Value	(95% CI) *p* Value	(95% CI) *p* Value
Child with disability in household (*N* = 6856)				
Yes	151/305 (49.51)	0.68 (0.54–0.86) *p* = 0.001	0.69 (0.55–0.86) *p* = 0.002	0.70 (0.55–0.89) *p* = 0.003
No	3863/6554 (58.94)	Ref.	Ref.	Ref.
District (*N* = 6856)				
Mukono	724/1176 (61.6)	1.54 (1.35–1.76) *p* < 0.001	1.51 (1.32–1.72) *p* < 0.001	1.75 (1.52–2.01) *p* < 0.001
Luwero	2050/4082 (50.2)	Ref.	Ref.	Ref.
Wakiso	1245/1684 (73.9)	2.83 (2.50–3.21) *p* < 0.001	2.73 (2.41–3.12) *p* < 0.001	2.84 (2.49–3.23) *p* < 0.001
Household size (*N* = 6856)				
	4019/6942 (57.9)	1.03 (1.01–1.05) *p* = 0.001	1.02 (1.00–1.04) *p* = 0.026	1.02 (1.00–1.04) *p* = 0.038
Child illness last 2 weeks (*N* = 6856)				
Yes	316/617 (51.2)	0.81 (0.73–0.90) *p* < 0.001	0.88 (0.79–0.98) *p* = 0.014	0.84 (0.75–0.94) *p* = 0.002
No	3703/6325 (58.6)	Ref.	Ref.	Ref.
Food assistance in last year (*N* = 6856)				
Yes	261/396 (65.9)	1.40 (1.13–1.73) *p* = 0.002	1.13 (0.91–1.42) *p* = 0.267	1.09 (0.86–1.36) *p* = 0.480
No	3754/6466 (58.1)	Ref.	Ref.	Ref.
Dietary adequacy (*N* = 6788)				
Yes	841/1186 (70.9)	1.88 (1.64–2.15) *p* < 0.001	2.02 (1.76–2.32) *p* < 0.001	1.85 (1.60–2.13) *p* < 0.001
No	3166/5607 (56.5)	Ref.	Ref.	Ref.
WASH score (*N* = 6854)				
	4019/6942 (57.9)	1.09 (1.07–1.11) *p* < 0.001	1.09 (1.08–1.11) *p* < 0.001	1.07 (1.05–1.09) *p* < 0.001

^a^ Adjusted for district, household size, child disability in the household, child illness in the last 2 weeks and food assistance in the last year.

^b^ Additionally adjusted for WASH score and dietary adequacy, based on three meals a day and consumption of five food groups in the last 7 days.

For households with a WASH score of 0, that is those with the lowest WASH adequacy, having a child with a disability was associated with 81% lower odds (OR = 0.19; 95% CIs: 0.06–0.57; *p* = 0.003) of having access to NaHS compared to households that did not have any children with disabilities (Table 4). Odds of access to NaHS for households with children with disabilities increased by 12% for every 1 unit increase in WASH score, with strong evidence of an interaction between having a child with disabilities and WASH score on the outcome of access to NaHS (interaction odds ratio; 1.12, 95% CIs: 1.02–1.22, *p* = 0.012). There was no evidence of interaction between households with children with disabilities and insufficient drinking water (*p* = 0.360), consuming food from all food groups in last week (*p* = 0.957) or children having three or more meals a day (*p* = 0.785) in their associations with access to NaHS (Table 4 ).

**Table 4:** Stratum‐specific odds ratios and test for interactions between households with a child with a disability and household access to nutrition and health services by drinking water sufficiently, WASH adequacy score, dietary intake and child having three meals a day.

	Stratum‐specific	95% CIs		
Characteristic^a^	Odds ratio	Lower	Upper	Interaction test *p* Values^a^
*N* = 6757				
Lacked sufficient drinking water last month				0.360
Yes	0.86	0.52	1.42	
No	0.65	0.50	0.86	
WASH adequacy score				0.012
0–20	1.12	1.02	1.22	
Consumed all food groups in last week				0.957
Yes	0.71	0.37	1.35	
No	0.70	0.54	0.90	
Children have 3+ meals a day				0.785
Yes	0.72	0.53	0.98	
No	0.67	0.46	0.99	

a From the likelihood ratio test.

## 4 DISCUSSION

This study describes access to nutrition and health services among households with and without children with disabilities in Uganda and explores interactions with WASH and dietary adequacy. We found that households with children have insufficient access to essential NaHS, especially for those households with children with disabilities. Overall, 42.1% of households reported not having access to all of the services they needed, of which 50.5% of households with children with disabilities and 41.1% of households without reported insufficient NaHS access. Currently in Uganda, only 72% of the population live within 5 km of a health facility, although this varies across regions (Odokonyero et al., 2017). Previous research by Odokonyero et al., found similar levels of difficulty in accessing NaHS in Uganda, especially for the poorest families and those living in rural areas (Odokonyero et al., 2017). In addition, we found evidence of an interaction between child disability and access to NaHS with WASH adequacy. Specifically, there is strong evidence that access to services for households with a child with a disability improved with increasing WASH adequacy. Of the households in our study, 57.9% reported having access to all of the NaHS they needed. This suggests above‐average access for SSA (42.6%) and for women in Uganda (40%) based on the latest Demographic and Health Survey (DHS) (Tessema et al., 2022; Uganda Bureau of Statistics—UBOS and ICF, 2018). As statistics on access to NaHS are difficult to obtain and there is a lack of common metrics for measuring access, especially for households with children with disabilities, our findings offer a useful estimate of the current situation and an indication that access to healthcare facilities in Uganda is insufficient (Dowhaniuk, 2021; Odokonyero et al., 2017) As there are many potential barriers for households to access NaHS, future research should explore barriers and facilitators for access, especially for individuals with disabilities.

### 4.1 Disability and health access

Of the households surveyed, 4.4% reported at least one child with a disability in the household, which is similar to the estimated prevalence of child disability in Uganda (United Nations Children's Fund [UNICEF], 2019). Insufficient access to NaHS is especially challenging for those with disabilities or households with children with disabilities who often face additional barriers and stigma around accessing health care (Adugna et al., 2020; Harrison et al., 2020). Our research found that access to NaHS was significantly worse for households with children with disabilities, with households having 30% lower odds of access to NaHS than households without children with disabilities even after adjusting for sociodemographic characteristics, WASH and dietary adequacy, which were used as markers of poverty. However, this could be an underestimate of the true prevalence due to factors such as stigma reducing reporting or lack of access to healthcare reducing diagnosis (Adugna et al., 2020; Engl et al., 2022; World Health Organization, 2011). This corroborates existing research that access to NaHS in SSA, and Uganda specifically, is worse for children with disabilities (Adugna et al., 2020; Harrison et al., 2020; Tessema et al., 2022). This is imapctful because we found households with children with disabilities experiencing a higher prevalence of illnesses, including diarrhea and skin infections. Article 25 of the United Nations Convention on the Rights of Persons with Disabilities sets out the rights of people with disabilities to receive high standards of equitable health care, free from discrimination and stigma but these standards can often be challenging to obtain when individuals face obstacles such as physical access or financial limitations (Adugna et al., 2020; United Nations, 2006). For example, in Ghana, households with children with cerebral palsy report favouring at‐home treatment only due to the high cost of medical treatment, caregiver burden and stigma associated with disability (Fonzi et al., 2021). Together, these findings highlight the magnitude of both the inequality and the need to improve accessibility of NaHS for children with disabilities.

### 4.2 Water access, sanitation and hygiene

Deworming and vaccinations were reported as the most important NaHS to respondents, yet these services were also reported as least accessible. According to the most recent Demographic and Health Survey (2016) in Uganda, 8.8% of children were infected with soil‐transmitted helminths (Uganda Bureau of Statistics—UBOS and ICF, 2018). Deworming pre‐school children is associated with reduced stunting, anaemia and malnutrition (Lo et al., 2018). Additionally, nearly a quarter of households surveyed report insufficient drinking water in the last month. Based on our WASH Adequacy score (12 out of 20 [IQR 10–14]), there is insufficient WASH for households with and without children with disabilities, with both experiencing similar WASH Adequacy (11.6 3.1% vs. 11.8 2.9%, *p* = 0.497). Although this is slightly better than the findings from the 2016 DHS in Uganda which found 32.5% of households with insufficient drinking water and 20% with an improved toilet, this still indicates a notable proportion of households lacking adequate safe drinking water, improved toilets or handwashing facilities, with likely serious implications on nutrition and health outcomes (Uganda Bureau of Statistics—UBOS and ICF, 2018). These findings support other research which indicates poverty, socioeconomic status, disability status and living in remote or rural areas are associated with insufficient WASH in Uganda (Enfield, 2018; United Nations Children's Fund [UNICEF], 2019; White et al., 2016).

### 4.3 Malnutrition and dietary adequacy

While 4488 (64%) of children older than 2 years old reportedly ate three meals a day, only 1196 (17.4%) achieved dietary adequacy in the previous week. This suggests that about a third of children do not have access to an adequate quantity of food, while nearly three quarters do not have access to an adequately diverse diet, with implications for undernutrition and particularly micronutrient deficiencies. After adjusting for sociodemographic factors, households whose children had dietary adequacy in the last week had two times the odds of access to NaHS than those without. Of the households with children with disabilities, 39.4% reported children not having three meals a day and 85.6% having an inadequate diet which was similar for households in these communities without children with disabilities. Few children younger than 5 years old (221 [2.6%]) were reported to have a MUAC below 12.5 cm, suggesting a low burden of wasting in this population, although the proportion of malnutrition was higher among households with children with disabilities (28 [6.3%] vs. 190 [2.4%], *p* < 0.001). Higher malnutrition among children with disabilities is congruent with similar findings by Rotenberg et al. (Rotenberg et al., 2024). The estimated prevalence of wasting is lower than the country average for children younger than 5 years old (3.7%). It is also in line with the downward trend in wasting observed throughout the last four Ugandan DHS reports, although future surveys and research should examine other proxy measures of malnutrition such low height‐for‐age, weight‐for‐height or weight‐for‐age (Uganda Bureau of Statistics—UBOS and ICF, 2018).

### 4.4 Strengths and limitations

The research utilized a large data set from a household survey with very little missing data. This research provides recent information on access to NaHS, WASH and nutrition in Uganda. The last published DHS is from 2016 and does not reflect the impact of the COVID‐19 pandemic (Uganda Bureau of Statistics—UBOS and ICF, 2018). However, the findings presented in this project must be considered in line with multiple limitations when interpreting results.

First, only the three districts of Wakiso, Luwero and Mukono were surveyed, thus the findings may not be representative of Uganda as a whole—it is possible there are additional notable regional differences in health, nutrition and access to services (Uganda Bureau of Statistics—UBOS and ICF, 2018). Additionally, the sample was unweighted, and information on probability of selection was not available. Therefore, those purposively sampled as Holt‐supported households are overrepresented and the non‐Holt supported households are underrepresented in the findings and information on response rate was not available. Systematic sampling was used to minimize selection bias as was training and supervision of surveyors, although all information was self‐reported by respondents on behalf of the household. The potential for responder bias should be taken into account when interpreting the findings.

This survey was conducted at the household level, therefore it was not possible to link any other information such as age or gender to the child with a disability, nor how many children in the household had a disability, but only to differentiate between households by whether any child had a disability. Disability status was self‐reported, future surveys should use a more standardized way of capturing functional difficulties in the communities, such as using the Washington Group Questionnaire (Washington Group on Disability Statistics, 2001). The prevalence within these communities of children with disabilities is likely higher than reported, as families may only report disability status if formally diagnosed.

There was the potential for measurement error during recording of survey responses, where some responses may have been written incorrectly, resulting in some implausible values. Measurement error could also have occurred when measuring children's MUAC, especially as there is limited guidance on applicability of MUAC use for children with disabilities (Hayes et al., 2023). Other anthropometric measurements were not taken limiting the full picture of the malnutrition situation in the region—wasting may be low but potentially other indicators such as stunting, may indicate a higher prevalence of malnutrition.

While efforts were made to adjust for potentially confounding factors, as much of the data was at the household level and not linkable to individuals, it was not possible to adjust for important confounding factors or such as age and gender, thus residual confounding should be taken into consideration when reviewing the outcomes. In the future, surveys should aim to use validated measures of dietary adequacy or WASH scores or explore other indicators of poverty, like household income, which was not measured in this survey. Due to the survey design, individual child MUAC measurements and disability status were not linked, and future research should investigate the relationship between MUAC measurements, disability and malnutrition (Hayes et al., 2023). Due to the nature of cross‐sectional studies, it was not possible to establish temporality with regard to exposure and outcome. It is likely that some of the disabilities reported, such as visual impairments, could have resulted from lack of access to NaHS or vitamin A supplementation. Therefore, the findings could present reverse causality from a bidirectional relationship, with poor access to NaHS leading to increased child disability as malnutrition and illness are major causes of disability. Those with poor WASH and dietary adequacy are therefore also more likely to develop a disability. This presents an important limitation of the interpretation. However, this does not impact the key message of the findings relating to the necessary improvement of access to NaHS both for children with disabilities and to prevent future illness and disability in general.

### 4.5 Recommendations

Future research should explore the needs of households with children with disabilities to improve access to essential services. Understanding the barriers and facilitators to full inclusion and ensuring all people's needs are met must be prioritized. Improving WASH facilities should be a key focus of NaHS for households with individuals with disabilities. Policy makers, government officials, local civil society organizations and NGOs should include those with disabilities in their planning and decisions. This research also identified that key practice and policy recommendations should ensure that deworming and vaccination services are included in NaHS, especially those targeting rural and hard‐to‐reach populations.

## 5 CONCLUSION

Substantial gaps remain in access to NaHS for households in Uganda, especially for households with children with disabilities. Access to essential services such as deworming and vaccinations falls short of family needs. There is also a need for increased WASH and nutrition services. Few children are receiving an adequately diverse diet, putting them at risk of malnutrition and micronutrient deficiencies. Access to NaHS is significantly lower for households with children with disabilities compared to those without, even after adjusting for sociodemographic factors, WASH and dietary adequacy. Households with children with disabilities and low WASH adequacy also have dramatically lower access to NaHS than those with high WASH adequacy. This highlights the challenges of poverty and rural residence and the need to improve access particularly for those with the fewest resources. Gaps in these essential services exacerbate inequalities and have implications for nutrition and health outcomes of children, families and communities. To better address the need for access to essential NaHS for families in Uganda, it remains vital that these services are inclusive, accessible and comprehensive.

## AUTHOR CONTRIBUTIONS

Study design was done by Isabel Rice, Emily DeLacey, Charles Opondo and Marko Kerac. Methods, data analysis, and quality control by Isabel Rice, Charles Opondo and Emily DeLacey. Data analysis and writing, original draft preparation by Isabel Rice, Charles Opondo and Emily DeLacey. Writing, review and editing by Isabel Rice, Charles Opondo, Marko Kerac, Lydia Nyesigomwe, Daniel Ekude, Julius Magezi, Andrew Kalanzi, Julia Hayes, Malia Robello, Sarah Halfman and Emily DeLacey All the authors have read and agreed to the published version of the manuscript.

## CONFLICT OF INTEREST STATEMENT

E. D., J. H., L. N., D. E., J. M., A. K., M. R. and S. H. work for Holt International.

## Supporting information

Supporting information.

Supporting information.

## Data Availability

Requests for access to this data need to be directed to Holt International. The data will be shared only on a contingent approval basis with interested parties. Additional related study protocols can be requested. Approval of a proposal, a data management protocol and a signed data access agreement will be required. To be addressed to: Holt International, info@holtinternational.org; 250 Country Club Road, Eugene, OR 97401; tel: 541.687.2202.

## APPENDIX A

Table A3 and A5.

**Table A1:** The PECO criteria outlining the population, exposure, comparator group and outcomes of the secondary data analysis (Mintzker et al., 2022).

PECO criteria	
Population	Households with children in Uganda from the districts of Mukono, Luwero and Wakiso
Exposure	Presence of at least one child with a disability within the household
Comparator	Households with no children with disabilities within the household
Outcomes	Household self‐reported access to nutrition and health services

**Table A2:** Nutrition, food access micronutrient supplementation, child illness and nutritional deficiency symptoms by household status.

Characteristic^a^	All households	Households with children with disabilities	Households without children with disabilities	*p* Value
*N* (%)	6942	305	6554	
School‐age children have school meals				*p* = 0.770
Yes	3440 (49.6)	150 (49.2)	3283 (50.1)	
Missing	77 (1.1)			
Household uses iodized salt				*p* = 0.003
Always	2528 (36.4)	137 (44.9)	2389 (36.5)	
Sometimes	4334 (62.4)	168 (55.1)	4164 (63.5)	
Missing	80 (1.2)			
Received food assistance in last year				*p* = 1.000
Yes	396 (5.7)	17 (5.6)	378 (5.8)	
Missing	80 (1.2)			
Children take any micronutrient supplements				*p* = 0.073
Yes	4120 (59.4)	168 (55.1)	3948 (60.2)	
Missing	80 (1.2)			
Motivation for giving supplements				*p* = 0.236
Prescribed by doctor	3079 (44.4)	146 (47.9)	2933 (44.8)	
Given by health profile	2619 (37.7)	102 (33.4)	2515 (38.4)	
Given by myself	123 (1.8)	8 (2.6)	114 (1.7)	
Given by Holt	1041 (15)	49 (16.1)	991 (15.1)	
Missing	80 (1.2)			
Which micronutrient/s children take				
Vitamin A	3863 (55.7)	155 (50.8)	3704 (56.5)	*p* < 0.001
Iron	58 (0.8)	2 (0.7)	56 (0.9)	p = 1.000
Zinc	140 (2)	13 (4.3)	127 (1.9)	*p* = 0.011
Calcium	110 (1.6)	3 (1)	107 (1.6)	*p* = 0.489
Folate	17 (0.2)	0	17 (0.3)	p = 1.000
Missing	80 (1.2)			
Any child took vitamin A in last 6 months				*p* = 0.585
Yes	4332 (62.4)	188 (61.6)	4142 (63.2)	
Missing	83 (1.2)			
Why child did not take vitamin A				*p* = 0.110
No access	6081 (87.6)	263 (86.2)	5816 (88.7)	
Chose not to	167 (2.4)	10 (3.3)	157 (2.4)	
Fear	12 (0.2)	2 (0.7)	10 (0.2)	
Not aware	599 (8.6)	30 (9.8)	569 (8.7)	
Missing	83 (1.2)			
Any child dewormed in last 6 months				*p* = 0.637
Yes	5126 (73.8)	232 (76.1)	4892 (74.6)	
Missing	83 (1.2)			
Why child was not dewormed				*p* = 0.73
No access	6400 (92.2)	284 (93.1)	6114 (93.3)	
Chose not to	97 (1.4)	4 (1.3)	93 (1.4)	
Fear	6 (0.1)	2 (0.7)	4 (0.1)	
Not aware	356 (5.1)	15 (4.9)	341 (5.2)	
Missing	83 (1.2)			
Any breastfed child				*p* = 0.894
Yes	1813 (26.1)	79 (25.9)	1734 (26.5)	
Missing	149 (2.2)			
Child younger than 5‐year‐old is Malnourished^a^				*p* < 0.001
Yes	221/8357 (2.6)	28/442 (6.3)	190/7785 (2.4)	
Number of meals a day for children				*p* = 0.088
One	314 (4.5)	20 (6.6)	294 (4.5)	
Two	2031 (29.3)	99 (32.5)	1931 (29.5)	
Three or more	4448 (64.1)	183 (60)	4264 (65.1)	
Missing	149 (2.2)			
Any child had an illness in the last 2 weeks				*p* = 0.001
Yes	4645 (66.9)	234 (76.7)	441 (67.3)	
Missing	83 (1.2)			
Illness type				
Diarrhoea	1236 (17.8)	70 (23)	1166 (17.8)	*p* = 0.015
Cough	3673 (52.9)	186 (61)	3487 (53.2)	*p* = 0.008
Fever	2561 (36.9)	149 (48.9)	2412 (36.8)	*p* < 0.001
Skin infection	1081 (15.6)	95 (31.2)	986 (15)	*p* < 0.001
Any child has nutritional deficiency symptoms				
Trouble seeing at night	231 (3.3)	43 (14.1)	188 (2.9)	*p* < 0.001
Goitre	70 (1)	7 (2.3)	63 (1)	*p* = 0.035
Bleeding gums	583 (8.4)	40 (13.1)	543 (8.3)	*p* = 0.006
Dental caries or tooth pain	1036 (14.9)	72 (23.6)	964 (14.7)	*p* < 0.001
Low energy or weakness	464 (6.7)	95 (31.2)	369 (5.6)	*p* < 0.001
Headaches	512 (7.4)	76 (24.9)	436 (6.7)	*p* < 0.001
Missing	84 (1.2)			

a Mid‐upper‐arm‐circumference (MUAC) less than 12.5 cm. (World Health Organization, 2019).

**Table A3:** Frequency of food group consumption in the last 7 days by children older than 2 years of age.

Characteristic	All households	Households with children with disabilities	Households without children with disabilities	*p* Value
*N* (%)	6942	305	6554	
Food group consumed in last 7 days				
Missing	149 (2.2)			
Meat or fish				*p* < 0.001
None	2966 (42.7)	148 (48.5)	2817 (43)	
1–2 days	2900 (41.8)	127 (41.6)	2772 (42.3)	
3–4 days	661 (9.5)	17 (5.6)	644 (9.8)	
5–6 days	165 (2.4)	5 (1.6)	160 (2.4)	
7 days	101 (1.5)	5 (1.6)	96 (1.5)	
Vegetables				*p* = 0.012
None	604 (8.7)	31 (10.2)	573 (8.7)	
1–2 days	2067 (29.8)	98 (32.1)	1968 (30)	
3–4 days	2195 (31.6)	103 (33.8)	2092 (31.9)	
5–6 days	1377 (19.8)	39 (12.8)	1338 (20.4)	
7 days	550 (7.9)	31 (10.2)	518 (7.9)	
Eggs				*p* = 0.465
None	4115 (59.3)	195 (63.9)	3919 (59.8)	
1–2 days	1921 (27.7)	76 (24.9)	1844 (28.1)	
3–4 days	442 (6.4)	15 (4.9)	427 (6.5)	
5–6 days	186 (2.7)	9 (3)	177 (2.7)	
7 days	129 (1.9)	7 (2.3)	122 (1.9)	
Fruit				*p* = 0.091
None	672 (9.7)	43 (14.1)	627 (9.6)	
1–2 days	1964 (28.3)	81 (26.6)	1883 (28.7)	
3–4 days	2064 (29.7)	90 (29.5)	1974 (30.1)	
5–6 days	1412 (20.4)	54 (17.7)	1358 (20.7)	
7 days	681 (9.8)	34 (11.2)	647 (9.9)	
Dairy				*p* = 0.38
None	2583 (37.2)	128 (42)	2453 (37.4)	
1–2 days	2273 (32.7)	111 (36.4)	2162 (33)	
3–4 days	857 (12.4)	28 (9.2)	829 (12.7)	
5–6 days	403 (5.8)	10 (3.3)	393 (6)	
7 days	677 (9.8)	25 (8.2)	652 (10)	

**Table A4:** Medical support for children with disabilities and disability prevalence.

District				
Characteristic	Mukono	Luwero	Wakiso	Total
*N* (%)	1176 (16.9)	4082 (58.8)	1684 (24.3)	6942 (100)
Child gets routine medical attention				
Children with disabilities in household	14 (29.2)	66 (34.4)	22 (33.9)	102 (1.5)
No Children with disabilities in household	71 (6.3)	141 (3.7)	1 (0.1)	213 (3.1)
Missing	0 (0)	66 (1.6)	17 (1)	83 (1.2)
Child receives adequate hospital support				
Children with disabilities in household	9 (18.8)	45 (23.4)	9 (13.9)	63 (0.9)
No children with disabilities in household	213 (18.9)	89 (2.3)	2 (0.1)	304 (4.4)
Missing	0 (0)	66 (1.6)	17 (1)	83 (1.2)
Disability prevalence				
*N* (%)	48 (4.1)	192 (4.7)	65 (3.9)	305 (4.4)
Missing	0 (0)	66 (1.6)	17 (1)	83 (1.2)
Type of disability (self‐described)				
ADD	2 (4.2)	10 (5.2)	2 (4)	14 (4.6)
Deaf	3 (6.3)	4 (2.1)	7 (10.8)	14 (4.6)
Limited hearing	8 (16.7)	10 (5.2)	2 (4)	20 (6.6)
Autism	1 (2.1)	4 (2.1)	0 (0)	5 (1.6)
Bi‐polar disorder	4 (8.3)	2 (1)	0 (0)	6 (2)
Blind	2 (4.2)	7 (3.7)	2 (4)	11 (3.6)
Multiple sclerosis	2 (4.2)	7 (3.7)	3 (4.6)	12 (3.9)
Chronic asthma	1 (2.1)	4 (2.1)	0 (0)	5 (1.6)
Visually impaired	3 (6.3)	11 (5.7)	8 (12.3)	22 (7.2)
Speech impediment	0 (0)	0 (0)	1 (1.5)	1 (0.3)
Down syndrome	3 (6.3)	7 (3.7)	1 (1.5)	11 (3.6)
Dyslexia	1 (2.1)	0 (0)	0 (0.0)	1 (0.3)
Epilepsy	2 (4.2)	3 (1.6)	0 (0.0)	5 (1.6)
Muscular dystrophy	1 (2.1)	11 (5.7)	1 (1.5)	13 (4.3)
Processing disorders	4 (8.3)	3 (1.6)	1 (1.5)	8 (2.6)
Oppositional defiance disorder	0 (0)	1 (0.5)	2 (4)	3 (1)
Lame	1 (2.1)	0 (0)	0 (0)	1 (0.3)
Sickle cell anaemia	0 (0)	1 (0.5)	1 (1.5)	2 (0.7)
Child has a feeding difficulty				
Yes	16 (1.4)	59 (1.5)	13 (0.8)	88 (1.3)
Missing	0 (0)	66 (1.6)	17 (1)	83 (1.2)

**Table A5:** Water access, sanitation and hygiene facilities by household status.

Characteristic	All households	Households with children with disabilities	Households without children with disabilities	*p* Value
*N* (%)	6942	305	6554	
Main source of drinking water				*p* = 0.637
Borehole water	4115 (59.3)	190 (62.3)	3924 (59.9)	
Bottled water	25 (0.4)	2 (0.7)	23 (0.4)	
Open well	787 (11.3)	30 (9.8)	757 (11.6)	
Spring water	454 (6.5)	18 (5.9)	436 (6.7)	
Tap water	1478 (21.3)	65 (21.3)	1411 (21.5)	
Missing	83 (1.2)			
Main source of water (cooking and washing)				*p* = 0.996
Borehole water	3760 (54.2)	170 (55.7)	3589 (54.8)	
Bottled water	41 (0.6)	1 (0.3)	40 (0.6)	
Open well	1080 (15.6)	49 (16.1)	1031 (15.7)	
Spring water	455 (6.6)	19 (6.2)	436 (6.7)	
Tap water	1523 (21.9)	66 (21.6)	1455 (22.2)	
Missing	83 (1.2)			
Lacked sufficient drinking water last month				*p* = 0.888
Yes	1531 (22.1)	66 (21.6)	1465 (22.4)	
Missing	112 (1.6)			
Water stored in containers with tight lids				*p* = 0.256
Yes	4133 (59.5)	174 (57.1)	3956 (60.4)	
Missing	83 (1.2)			
Water safety method				*p* = 0.424
Boiling	4104 (59.1)	181 (59.3)	3920 (59.8)	
Chemical agents	17 (0.2)	0	17 (0.3)	
Filtering system	68 (1)	6 (2)	62 (1)	
None	2670 (38.5)	118 (38.7)	2552 (38.9)	
Missing	83 (1.2)			
Daily handwash frequency				*p* = 0.001
None	326 (4.7)	30 (9.8)	296 (4.5)	
1–2 times	1532 (22.1)	55 (18)	1476 (22.5)	
3–4 times	2129 (30.7)	125 (41)	2745 (41.9)	
More than five times	2129 (30.7)	95 (31.2)	2034 (31.1)	
Missing	83 (1.2)			
Has latrine with roof				*p* = 0.480
Yes	3817 (55)	176 (57.7)	3639 (55.5)	
Missing	83 (1.2)			
Latrine cleaning frequency				*p* = 0.302
Never	742 (10.7)	42 (13.8)	700 (10.7)	
After 2 days	2039 (29.4)	93 (30.5)	1945 (29.7)	
Every other day	1429 (20.6)	56 (18.4)	1372 (20.9)	
Daily	2649 (38.2)	114 (37.4)	2534 (38.7)	
Missing	83 (1.2)			
Type of toilet				*p* = 0.368
Pit latrine	5126 (73.8)	221 (72.5)	4902 (74.8)	
Ventilated pit latrine	588 (8.5)	26 (8.5)	562 (8.6)	
Raised pit latrine	671 (9.7)	34 (11.2)	637 (9.7)	
Public toilet	243 (3.5)	10 (3.3)	233 (3.6)	
None	208 (3)	11 (3.6)	197 (3)	
Other	23 (0.3)	3 (1)	20 (0.3)	
Missing	83 (1.2)			

Directed acyclic graph (DAG) causal pathway exposure, outcome, and confounding factors.

A directed acyclic graph was used to explore the association between the exposure (children with disabilities in the household) and outcome (access to nutrition and health services) alongside potentially confounding factors to inform adjustment of the regression models.

The following factors were deemed to be potentially confounding:

1. District
2. Household size
3. Child illness in last 2 weeks
4. Household received food assistance in the last year
5. Access to WASH
6. Dietary adequacy

The following factors were deemed to be on causal pathway of the association between the main exposure and outcome:

1. Holt‐supported household, which would increase access to NaHS
2. Marital status, as this may be affected by having a child with disabilities, and single or widowed people may have less access to NaHS than married couples
3. School meals and school health screening, as children with disabilities may be less likely to attend school, which would reduce access to NaHS at school, and reduce dietary adequacy, which would be associated with NaHS as it is a marker for poverty
4. Miscarriage in the last 5 years and losing a child younger than 5‐year‐old; both may be on the reverse causal pathway, with poor access to NaHS leading to this outcome, which is also associated with greater likelihood of child disability

*Interactions*

Information on WASH and dietary adequacy of children were tested for interaction with the association between the main exposure and outcome. These were selected as proxy markers of poverty as there was no data available relating to financial or socioeconomic status, to assess whether better dietary adequacy and WASH (therefore lower poverty) would alter barriers faced to accessing nutrition and health services.
