# Supplementary material for: Children with disabilities lack access to nutrition, health and WASH services: A secondary data analysis
Source: Matern Child Nutr. 2024 Apr 2;20(3):e13642. doi: 10.1111/mcn.13642 (PMC11168356; doi:10.1111/mcn.13642)
Supplement: Supplementary file 2 — Supporting information. [file MCN-20-e13642-s002.pdf]

NUTRITION AND HEALTH HOUSEHOLD SURVEY - UGANDA

Purpose
This household survey is undertaken by Holt International and Holt Uganda. Holt International implements strong monitoring and evaluation processes in all countries where Holt works. This household survey is intended to capture health and nutrition-related information within communities served by Holt to inform program development and track relevant community health indicators.

Voluntary Participation and Withdrawal from the Program
Your participation is purely voluntary. If you decide to participate in this survey, we will ask you to sign the consent form below. You may refuse to participate or withdraw from the program at any time without stating a reason. Such a decision will not affect your relationship with us, either now or in the future.

What does agreeing to participate mean for me?
Participation involves answering survey questions related to the health and nutrition status of you and the members of your household. This survey is expected to last 15-20 minutes.

Risks and Discomforts
No risks are expected to be encountered by you however you may find some of the questions to be sensitive.

Compensation
There will be no remuneration for participants of this survey.

Confidentiality and Data Use
No names or identifying information will be included in any use of these data and your responses to this survey will remain confidential. Data collection will be conducted by both Holt staff and third-party community health workers. Data collected will be electronically recorded and stored securely by Holt International. All de-identified data is used by Holt International for reporting, program audits, publications, presentations and other activities used to raise awareness about the needs of programs and people Holt International works with.

Signed Consent

Thank you for taking time to read or listen to the content of this information sheet. Should you like any further information or would like to withdrawal your response to this survey after completion, please reach out to us using the following contact details:

Name, Title, Holt Uganda

Phone Number

By signing below, you are stating you have read or been read the above information and voluntarily agree to participate in this survey.

Participant Signature ________________________________ Date________ /________ /_________

Time Interview Started_____________

Statement by the Person Taking Consent:

I have accurately read out the Statement of Consent to the potential participant, and to the best of my ability made sure that the participant understands that they have the choice of whether to participate in the study.

I confirm that the participant was given an opportunity to ask questions about the study, and all the questions asked by the participant have been answered correctly and to the best of my ability. I confirm that the individual has not been coerced into giving consent, and the consent has been given freely and voluntarily.

Name of Team Leader : ____

Name of Surveyor : ____

Sign : ____

<u>Note:</u> Confidentiality will be guaranteed

HOLT NUTRITION ASSESSMENT

A. Area Identification					
District		Village / Community		Zone	
Sub County			**Household Number***		

B. Biography of Respondent						
Sex of Respondent	0	Female	1	Male		
Status of Marriage	0	Married	1	Single	2	Widow

C. Household Information							
Household Type	0	Husband & Wife	1	Headed by Grandmother	2	Female Headed	
Number of people living in the household	Adults >30 years		Btwn 13 - 18 years		Btwn 1 – 2 yrs		
	Adults 19 – 29 yrs		Btwn 3 – 12 yrs		Below 12 months		
Are there any children with disabilities or special needs living within the household?	0	No	1	Yes			
Have you ever lost a child below 5 years old in this household?	0	No	1	Yes			
If yes, please include year.							

D. Access to Services while at home				
Have you ever attended a Holt organized Health Camp?	0	No	1	Yes
In case of sickness do you visit a health Center?	0	No	1	Yes
	2	Self-medicate		
What health services are most important to you?	0	Vitamin supplements		
	1	Deworming		
	3	Child growth screening		
	4	Wellness check		
	5	Vaccinations		
	6	Prenatal care		
	7	Health & nutrition education		
Do you currently have access to all health services that you need?	0	No	1	Yes
	0	Vitamin supplements		
	1	Deworming		
	2	Child growth screening		
	3	Wellness check		

If no, which ones do you **not** have access to?	4	Vaccinations
	5	Prenatal care
	6	Health & nutrition education
	7	Other:

E. Access to Services while at school				
Have all children in this household had a medical checkup within past one year	0	No	1	Yes
Do school children receive health screening or services at school?	0	No	1	Yes
Do school children participate in a school meal program?	0	No	1	Yes
If no, how does the child find what to eat while at school?				
Do you have any additional comments or concerns regarding your household's access to services? If yes, please describe.				

F. Nutrition and Health

Child 1.	Name of Child			Sex	0	Male	
					1	Female	
Age of the Child	0 – 12 Months		Btwn 3 – 5 years		Btwn 13 - 19 yrs		
	13– 36 Months		Btwn 6 - 12 yrs				

If possible, measure and indicate the following:	Weight of the Child	
	Height of the Child	
	BMI of the child (calculate)	

For children 6 months - ≤5 years		
Obtain and record child's MUAC measurement.		cm
Record the child's MUAC measurement here:	RED (Less than 11.5 cm)	*(Tick)*
	YELLOW (Between 11.5 and 12.4 cm)	*(Tick)*
	GREEN (Greater than or equal to 12.5 cm)	*(Tick)*

Child 2.	Name of Child			Sex	0	Male	
					1	Female	
Age of the Child	0 – 12 Months		Btwn 3 – 5 years		Btwn 13 - 19 yrs		
	13– 36 Months		Btwn 6 - 12 yrs				

	Weight of the Child	

HOLT NUTRITION ASSESSMENT

**If possible**, measure and indicate the following:	Height of the Child	
	BMI of the child (calculate)	

For children 6 months - ≤5 years		
Obtain and record child's MUAC measurement.		cm
Record the child's MUAC measurement here:	RED (Less than 11.5 cm)	*(Tick)*
	YELLOW (Between 11.5-12.4 cm)	*(Tick)*
	GREEN (Greater than or equal to 12.5 cm)	*(Tick)*

Child 3.	Name of Child		Sex	0	Male	
				1	Female	

Age of the Child	0 – 12 Months		Btwn 3 – 5 years		Btwn 13 - 19 yrs	
	13– 36 Months		Btwn 6 - 12 yrs			

**If possible**, measure and indicate the following:	Weight of the Child	
	Height of the Child	
	BMI of the child (calculate)	

For children 6 months - ≤5 years		
Obtain and record child's MUAC measurement.		cm
Record the child's MUAC measurement here:	RED (Less than 11.5 cm)	*(Tick)*
	YELLOW (Between 11.5 and 12.4 cm)	*(Tick)*
	GREEN (Greater than or equal to 12.5 cm)	*(Tick)*

Child 4.	Name of Child		Sex	0	Male
				1	Female

Age of the Child	0 – 12 Months		Btwn 3 – 5 years		Btwn 13 - 19 yrs	
	13– 36 Months		Btwn 6 - 12 yrs			

**If possible**, measure and indicate the following:	Weight of the Child	
	Height of the Child	
	BMI of the child (calculate)	

For children 6 months - ≤5 years		
Obtain and record child's MUAC measurement.		cm
Record the child's MUAC measurement here:	RED (Less than 11.5 cm)	*(Tick)*
	YELLOW (Between 11.5 and 12.4 cm)	*(Tick)*
	GREEN (Greater than or equal to 12.5cm)	*(Tick)*

Child 5.	Name of Child		Sex	0	Male

						1	Female
Age of the Child	0 – 12 Months		Btwn 3 – 5 years		Btwn 13 - 19 yrs		
	13– 36 Months		Btwn 6 - 12 yrs				
**If possible**, measure and indicate the following:	Weight of the Child						
	Height of the Child						
	BMI of the child (calculate)						
For children 6 months - ≤5 years							
Obtain and record child's MUAC measurement.							cm
Record the child's MUAC measurement here:	RED (Less than 11.5 cm)						*(Tick)*
	YELLOW (Between 11.5 and 12.4 cm)						*(Tick)*
	GREEN (Greater than or equal to 12.5 cm)						*(Tick)*

Child 6.	Name of Child			Sex		0	Male
						1	Female
Age of the Child	0 – 12 Months		Btwn 3 – 5 years		Btwn 13 - 19 yrs		
	13– 36 Months		Btwn 6 - 12 yrs				
**If possible**, measure and indicate the following:	Weight of the Child						
	Height of the Child						
	BMI of the child (calculate)						
For children 6 months - ≤5 years							
Obtain and record child's MUAC measurement.							cm
Record the child's MUAC measurement here:	RED (Less than 11.5 cm)						*(Tick)*
	YELLOW (Between 11.5 and 12.4 cm)						*(Tick)*
	GREEN (Greater than or equal to 12.5 cm)						*(Tick)*

Child 7.	Name of Child			Sex		0	Male
						1	Female
Age of the Child	0 – 12 Months		Btwn 3 – 5 years		Btwn 13 - 19 yrs		
	13– 36 Months		Btwn 6 - 12 yrs				
**If possible**, measure and indicate the following:	Weight of the Child						
	Height of the Child						
	BMI of the child (calculate)						

For children 6 months - ≤5 years		
Obtain and record child's MUAC measurement.		cm
Record the child's MUAC measurement here:	RED (Less than 11.5 cm)	*(Tick)*
	YELLOW (Between 11.5 and 12.4 cm)	*(Tick)*
	GREEN (Greater than or equal to 12.5 cm)	*(Tick)*

Child 8.	Name of Child				Sex	0	Male
						1	Female
Age of the Child	0 – 12 Months		Btwn 3 – 5 years		Btwn 13 - 19 yrs		
	13– 36 Months		Btwn 6 - 12 yrs				
**If possible**, measure and indicate the following:	Weight of the Child						
	Height of the Child						
	BMI of the child (calculate)						

For children 6 months - ≤5 years		
Obtain and record child's MUAC measurement.		cm
Record the child's MUAC measurement here:	RED (Less than 11.5 cm)	*(Tick)*
	YELLOW (Between 11.5 and 12.4 cm)	*(Tick)*
	GREEN (Greater than or equal to 12.5 cm)	*(Tick)*

Child 9.	Name of Child				Sex	0	Male
						1	Female
Age of the Child	0 – 12 Months		Btwn 3 – 5 years		Btwn 13 - 19 yrs		
	13– 36 Months		Btwn 6 - 12 yrs				
**If possible**, measure and indicate the following:	Weight of the Child						
	Height of the Child						
	BMI of the child (calculate)						

For children 6 months - ≤5 years		
Obtain and record child's MUAC measurement.		cm
Record the child's MUAC measurement here:	RED (Less than 11.5 cm)	*(Tick)*
	YELLOW (Between 11.5 and 12.4 cm)	*(Tick)*
	GREEN (Greater than or equal to 12.5 cm)	*(Tick)*

Child 10.	Name of Child		Sex	0	Male

					1	Female
Age of the Child	0 – 12 Months		Btwn 3 – 5 years		Btwn 13 - 19 yrs	
	13– 36 Months		Btwn 6 - 12 yrs			

**If possible**, measure and indicate the following:	Weight of the Child	
	Height of the Child	
	BMI of the child (calculate)	

For children 6 months - ≤5 years		
Obtain and record child's MUAC measurement.		cm
Record the child's MUAC measurement here:	RED (Less than 11.5 cm)	*(Tick)*
	YELLOW (Between 11.5 and 12.4 cm)	*(Tick)*
	GREEN (Greater than or equal to 12.5 cm)	*(Tick)*

G. Access to Micronutrients / Supplements				
Do you cook with iodized salt?	0	Always		
	1	Sometimes		
	2	Never		
	3	I don't Know		
Has the family received food assistance in the last one year?	0	No	1	Yes
If yes, describe the food assistance provided:				
Do you give your child any multi-vitamin syrup or capsule?	0	No	1	Yes
If yes, list the multi-Vitamins you give.	0	Vitamin A		
	1	Iron		
	2	Zinc		
	3	Calcium		
	4	Folate		
	5	Other:		
What is the reason to give?	0	Prescribed by a Doctor		
	1	Provided by the Clinic		
	2	Given by myself		
	3	Routine Service (Holt)		

H. Deworming and Vitamin A					
For Children below 59 months			For children 1 to 12 years		
Did your child / children take Vitamin A supplement within the past six (6) months?	0	No	Did your child / children take deworming treatment within the past six (6) months?	0	No
	1	Yes		1	Yes
	0	No access		0	No access

If no, why? (*Tick*)	1	Chose not to	If no, why? (*Tick*)	1	Chose not to
	2	Fear		2	Fear
	3	Not aware		3	Not aware

I. Intake

For a child below 24 months			For children 2 to 17 years		
Do you have a child who is breastfed?	0	Yes	How many meals a day does your child eat?	0	Three meals
	1	No		1	Two Meals
				2	One Meal
If Yes, when do you expect to stop?	0	At six months	How many days has your child eaten meat / fish in the last seven (7) days?	0	None
	1	At 12 months		1	1 to 2 days
	2	Beyond 13 months		2	3 to 4 days
				3	5 to 6 days
				4	7 days

For a children 2 to 18 Years					
How many days has your child eaten vegetables in the last seven (7) days?	0	None	How many days has your child eaten eggs in the last seven (7) days?	0	None
	1	1 to 2 days		1	1 to 2 days
	2	3 to 4 days		2	3 to 4 days
	3	5 to 6 days		3	5 to 6 days
	4	7 days		4	7 days
How many days has your child eaten fruits in the last seven (7) days?	0	None	How many days has your child eaten dairy products in the last seven (7) days?	0	None
	1	1 to 2 days		1	1 to 2 days
	2	3 to 4 days		2	3 to 4 days
	3	5 to 6 days		3	5 to 6 days
	4	7 days		4	7 days

J. Overall Child’s Health

Has any child had diarrhea in the last two weeks?	0	No	1	Yes
Has any child had an illness with cough in the last two weeks?	0	No	1	Yes
Has any child had fever in the last two weeks?	0	No	1	Yes
Has any child had a skin infection within the last month?	0	No	1	Yes

K. Deficiency Assessment

Does any child have trouble seeing at night?	0	No	1	Yes
Does any child have a goiter?	0	No	1	Yes

(Goiter is an enlargement of the thyroid gland visible as a swelling of the front of the neck)				
Does any child have gums that bleed?	0	No	1	Yes
Does any child have dental carries or teeth that hurt?	0	No	1	Yes
Does any child have low energy / fatigue / weakness?	0	No	1	Yes
Does any child experience confusion, apathy, lack of energy, low mood, irritability or headaches?	0	No	1	Yes
Do you have any additional comments or concerns regarding children's overall health? If yes, please describe.				

L. Only for Respondents of Households with Children with Disabilities or Special Needs				
What type of disability does your child have?				
How does it affect their daily functioning?				
Does your child with a special need have difficulty eating?	0	No	1	Yes
If yes, how do you ensure that s/he is eating sufficient meals daily to avoid becoming malnourished?				
Do you take him / her for routine medical attention?	0	No	1	Yes
Do you feel you and your child are getting proper support from the hospital?	0	No	1	Yes
If not, what kind of help does your child need medically?				
What kind of exercises does s/he do for physical fitness?				

M. WASH					
What is the main source of drinking water?	0	Borehole water	What is the main source of water for cooking and washing?	0	Borehole water
	1	Bottled water		1	Bottled water
	2	Open well		2	Open well
	3	Spring water		3	Spring water
	4	Tap water		4	Tap water

In the last one month, has there been a time when you did not have sufficient water for drinking?	0	No	1	Yes
Is water stored in containers with tight fitted lids?	0	No	1	Yes

What do you usually do	0	Boiling	How many times a day do	0	0 times
	1	Chemical agents		1	1 to 2 times

to make water safe to drink?	2	Filtering system	you wash your hands?	2	3 to 4 times
	3	None		3	More than 5 times

Do you have a functional toilet / latrine (with roof and door)?	0	No	1	Yes		
How often do you clean your latrine facility?	0	Daily	1	Every other day	2	Weekly
	3	Never	4	Other		
What kind of toilet facility do you have?	0	Pit Latrine				
	1	Ventilated Pit Latrine				
	2	Raised Pit Latrine				
	3	Public Toilet				
	4	None				
	5	Other:				

THANK YOU FOR YOUR TIME

Time interview ended _________
